# Graph-Aware AURALSTM: An Attentive Unified Representation Architecture with BiLSTM for Enhanced Molecular Property Prediction

**DOI:** 10.1007/s11030-025-11197-4

**Published:** 2025-04-25

**Authors:** Muhammed Ali Pala

**Affiliations:** 1 Department of Electrical and Electronics Engineering, Faculty of Technology, Sakarya University of Applied Sciences, 54050 Sakarya, Turkey; 2 Biomedical Technologies Application and Research Center (BIYOTAM), Sakarya University of Applied Sciences, Sakarya, Turkey

**Keywords:** Molecular property prediction, Graph neural networks, BiLSTM, Deep learning, Drug design and discovery

## Abstract

Predicting molecular properties with high accuracy is essential across scientific fields, from drug discovery and biotechnology to materials science and environmental research. In biomedical sciences, accurate molecular property prediction is crucial for elucidating disease mechanisms, identifying potential drug candidates, and optimising various processes. However, existing approaches, often based on low-dimensional representations, fail to capture the intricate spatial and structural complexities of molecular data. This study introduces a novel hybrid deep learning model, the Graph-Aware AURA-LSTM (Attentive Unified Representation Architecture—Long Short-Term Memory), designed to determine molecular properties with unprecedented accuracy using advanced graphical representations. AURA-LSTM combines multiple Graph Neural Network (GNN) architectures, specifically Graph Convolutional Networks (GCNs), Graph Attention Networks (GATs), and Graph Isomorphism Networks (GINs), in a parallel structure to comprehensively capture the multidimensional structural features of molecules. Within this architecture, GCNs incorporate local structural relationships, GATs apply attention mechanisms to highlight critical structural elements, and GINs capture intricate molecular details through isomorphic distinction, resulting in a richly detailed feature matrix. The feature layer then processes this BiLSTM matrix, which evaluates temporal relationships to enhance molecular feature classification. Evaluated on eight benchmark datasets, AURA-LSTM demonstrated superior performance, consistently achieving over 90% accuracy and outperforming state-of-the-art methods. These results position AURA-LSTM as a robust tool for molecular feature classification, uniquely capable of integrating temporally aware insights from distinct GNN architectures.

## Introduction

In today’s rapidly advancing science and technology, understanding and predicting the properties of molecules, the basic building blocks of matter, are vital for many disciplines. Prediction of the properties of existing molecules studied in fields such as chemistry, materials science, nanotechnology and energy research, and design and development of innovative molecules are based on accurate modelling of interactions and properties at the molecular level [1–4]. Especially in biomedical sciences, it is crucial for laboratory and clinical studies in areas such as developing effective drugs against diseases and repositioning drugs [5, 6]. In personalised medicine, identifying disease-specific biomarkers and optimising treatment strategies based on patient’s genetic profiles are based on accurate molecular property predictions [7–10]. Moreover, understanding disease mechanisms and identifying new therapeutic targets requires the study of complex biomolecular processes, such as protein–protein interactions, protein folding and enzyme kinetics [11]. Modelling and analysis of these complex biological systems require computational methods [11, 12]. In this context, knowledge of the physicochemical properties of molecules, such as solubility, melting point and reactivity and their biological activities, such as toxicity and binding affinity, plays a critical role in the development of new drugs, catalysts and functional materials [13–15]. Traditionally, these properties of molecules are determined experimentally. However, experimental determination of these properties is costly, time-consuming and requires high purity and a large quantity of samples. Experimental methods may sometimes be impossible [16, 17]. This situation constitutes a severe bottleneck today when the number of potential molecular candidates planned to be used in various fields rapidly increases. Therefore, severe theoretical and practical limitations are encountered, especially in cases where many molecules need to be screened and analysed.

There are many computational methods to overcome the limitations in molecular property identification processes. These methods are realised by representing molecules differently and extracting features from these representations. These computational methods allow molecules to be represented in 1D, 2D or 3D space [18–20]. A standard method that will enable molecules to be represented in 1D is the Simplified Molecular Input Line Entry System (SMILES) representation. SMILES encodes molecules as one-dimensional text strings for substructure searches, similarity measurements and rule-based predictions [21]. The presence or absence of specific functional groups in the SMILES string of a molecule allows information to be obtained about whether they affect a particular property of the molecule. Another necessary type of representation is the representation of molecules as graphical representations, which is a non-grid data form [22, 23]. Molecular graphs are an essential representation method that encode the structure of molecules in nature in two dimensions by representing atoms as nodes and bonds as edges, and this representation has a very high generalisation capability as it visualises all aspects of molecular structure. Graphical representations are particularly effective in capturing interactions and structural similarities between molecules more accurately, allowing more information to be collected than classical vector representations [24, 25]. Therefore, these graphical representations are a valuable data source for analysing intermolecular relationships and learning these relationships through models. The detailed information provided by molecular graphs has paved the way for developing innovative computational methods rather than traditional ones. In particular, graph-based deep learning methods make it possible to predict chemical properties by learning molecules’ atomic structures and bonds, modelling molecular activities, and making more precise predictions in applications such as drug discovery [26–28]. In this context, deep learning models such as GNNs have the potential to perform detailed analyses at the molecular level by capturing complex and multidimensional relationships between molecules.

GNNs are powerful deep learning models that provide a unique solution, especially for graph data problems. GNNs, which are frequently used in the analysis of molecular structures, can learn and infer chemical structures in all aspects by treating atoms in molecules as nodes and bonds as edges. This allows for a more accurate prediction of molecules’ physical and chemical properties and the modelling of complex molecule interactions [29–31]. One of the main advantages of GNNs is their ability to handle variable dimensional molecules. Without reducing to fixed dimensional vectors such as SMILES strings or derived fingerprints, GNNs can work directly on molecular graphs [32]. In this respect, GNNs prevent the loss of structural information and allow more accurate molecular feature predictions. Furthermore, GNNs can capture long-range dependencies in molecular graphs, which is especially important for large and complex molecules, such as proteins or polymers. The flexible architecture of GNNs allows them to be adapted to different molecular feature prediction tasks. These models handle the multidimensional networks of relationships presented by graphical data, minimising information loss and enriching deep learning processes with the detailed information provided by graphical representations.

The diversity of GNN types has been developed to analyse the complex structural properties of graph data from different perspectives and to provide optimal performance in specific problem types. GCNs, GATs and GINs are the most common and advanced GNN architectures. These architectures are specialised to address the diverse analysis needs inherent in graph data structures, and each offers advantages specific to particular problems [33, 34]. GCNs update node properties in graphs with local neighbourhood information, allowing a local transfer of information for each node. Principally, the graph allows the propagation of node features using the convolution operator. Using a weighted average, this operator combines the features of each node with those of its neighbours. This feature makes it possible to obtain efficient results in densely neighbouring graphs; hence, GCNs are particularly preferred when it is necessary to reduce the data size and optimise the computational cost. Therefore, GCNs allow high-performance results from densely neighbouring graph data. However, the fixed weight of the information that GCNs receive from neighbouring nodes causes the operator to affect all nodes to the same degree [35]. In this respect, it may limit flexibility in problems requiring selective information transfer. GAT architectures have been developed to eliminate this problem. GATs are architectures that try to overcome the problem of GCNs using fixed weights by using an attention mechanism. In other words, they assign different weights to each neighbour of a node to process other critical structural features within the molecule. This feature makes it possible to change the information transfer between nodes dynamically. For this reason, it shows higher performance, especially in more complex and heterogeneous graph data. The attention mechanism of GATs provides a flexible flow of information according to the node neighbourhood, allowing the model to provide a more flexible and high learning capability [36]. GINs, another important GNN architecture, have been developed to accurately distinguish isomorphic graph structure patterns. GINs enable more in-depth analysis of node and edge features, especially in cases where structural similarities between graphs are high. In this respect, they learn the distinguishing features of graph structures more effectively detect subtle structural differences between graphs, enabling highly successful results, especially in classification tasks. In particular, the ability of GINs to capture isomorphic structures plays a critical role in situations where graph data are very similar but have minor differences. While the theoretical strength of GINs is based on their ability to distinguish graph isomorphism, this can be a disadvantage in practice. This can make GINs sensitive to small changes in graph structure. Therefore, this may lead to a decline in generalisation performance when working with noisy or incomplete data. In conclusion, these three different GNN architectures have been developed for modelling the multidimensional and relational structure of graph data; GCNs provide efficient information transfer in simple and dense neighbourhood relationships, GATs provide selective and importance-based information transfer with the attention mechanism and GINs provide superiority in the decomposition of graph structures. In particular, GCNs are generally effective in static graphics, while GATs and GINs can operate in both spatial and temporal space.

## Related works

Traditional machine learning, especially deep learning methods, has been widely used to predict molecular features. Hybrid approaches combining different data forms enable the development of innovative and accurate techniques. In particular, GNNs and their hybrids are promising for classifying and understanding graph-structured molecules. These methods are used for the classification of molecular properties and the discovery of new molecular structures. There are several methods in the literature, some focussing on a single molecular form, while others combine different forms of data to evaluate molecules more comprehensively.

A common limitation of graph-based deep learning methods is their tendency to overlook the hierarchical structure inherent in molecules and rely primarily on feature-based inference. To address this deficiency, the Hierarchical Informative Graph Neural Network (HiGNN) was proposed [37]. HiGNN allows the combination of molecular graphs and retrosynthetically interesting chemical substructure fragments with message-passing mechanisms. In this way, they uncovered a molecule-part mechanism to improve the interpretability of the model. Furthermore, many existing deep learning models for molecular feature prediction suffer from limitations in atom-level feature extraction and often neglect the significant influence of substructures on molecular properties. To address this problem, the Hierarchical Molecular Graph Neural Network (HimGNN) provides a new framework that combines atom- and motif-level information using the hierarchical structure of molecules [38]. HimGNN learns hierarchical topology representations using graph neural networks in atom-based and motif-based graphs and enriches Motif-MPNN with a Transformer-based local boosting module. Furthermore, a contextual self-scaling module is integrated to model the dependencies between atom and motif properties. Deep learning models frequently focus on molecular representation data from a single perspective, potentially limiting their predictive power for complex tasks. Seeking to provide a more versatile molecular representation, the Deep Learning Framework for Multi-Type Feature Fusion (DLF-MFF) integrates four features: 1D molecular fingerprints, 2D molecular graphs, 3D molecular graphs and molecular images [20]. These features are processed by FCNN, GCN, Equivariant Graph Neural Networks (EGNN) and Convolutional CNN, combined into a fusion layer, and fed into a prediction layer for final prediction. A potential vulnerability in many deep learning methods lies in the common practice of initialising trainable parameters with random values, which may not always lead to optimal convergence or robust performance, especially in complex tasks like drug-target affinity prediction. The proposed ColdDTA model aims to mitigate challenges related to model initialisation and generalisation through attention-based feature fusion following data augmentation [39]. The ColdDTA method generates new drug-target pairs by subgraphing the drugs and aims to model these interactions more effectively using an attention mechanism. The model uses GNN for drug feature prediction and convolutional neural networks for protein feature prediction. Recognising the limitations inherent in relying on single molecular representations and the need for improved generalisation, PremuNET was developed for molecular feature prediction, aiming to eliminate various problems with its multi-representation fusion network approach [40]. PremuNet consists of two main structures: PremuNet-L, which uses SMILES arrays, molecular fingerprints and Transformer-based GNN models to process low-dimensional features, and PremuNet-H, a pre-trained GNN model combining 2D and 3D information for high-dimensional features. Both structures are pre-trained with self-supervised learning methods to improve performance. Thanks to this multi-representation strategy, learning the representation forms of molecules such as fingerprints, SMILES sequences, and 2D and 3D structures simultaneously and improving task performance is possible. Existing low-dimensional molecular representations often prove inadequate for capturing the full complexity, particularly spatial information, required for accurate molecular feature prediction. To address the inadequacy of these representations, AEGNN-M, a novel 3D graph-spatial matching model that combines 2D connectivity information with 3D spatial information, is developed [41]. This model aims to build a comprehensive representation of the molecule that encompasses connectivity and geometric properties using a Graph GAT for molecular graph representation and an EGNN for processing 3D spatial coordinates. By combining the features obtained from GAT and EGNN, this representation aims to overcome classification and regression problems. While various computational methods, such as quantitative structure–activity relationships, are widely used, they generally require extensive feature engineering or high computational capability. Furthermore, relying on single modalities like SMILES or graphs alone can limit the captured information. To overcome these problems, MMSG has been developed to combine multi-modal information from SMILES sequences of molecules and molecular graphs [42]. Combining the advantages of different molecular representations, this framework utilises a modified self-attention mechanism to capture hidden bond-level information in SMILES arrays. Furthermore, a Bidirectional Message Passing Graph Neural Network method is developed to enhance the information flow between atoms and bonds. This method enables more efficient message interactions through customised operators for nodes and edges and bidirectional message passing. Seeking to improve the accuracy and reliability of molecular feature prediction beyond what is achievable with fingerprint or graph-based methods alone, a novel deep learning model, FGNN, which combines the advantages of fingerprint and graph-based representations, is developed [43]. The FGNN consists of two basic sub-networks: FPNN, which processes molecular fingerprints and achieves dimensionality reduction with the Maximum Relevance Minimum Redundancy feature selection algorithm, and a GNN that uses GAT and Graph Collaborative Information (GCI) layers to extract structural information from molecular graphs. While GAT captures interactions between pairs of atoms, it may struggle with interactions beyond direct covalent bonds; the GCI layer specifically overcomes this limitation by allowing the modelling of the collective effect of neighbouring groups of atoms on each atom, including non-covalent interactions. The proliferation of diverse computational methods presents a challenge for researchers seeking to apply and compare them readily. To facilitate the application of all these different methods, a platform called MalariaFlow has been developed [44]. The platform combines several machine learning algorithms such as Random Forest, XGBoost, Attentive FP, GAT, GCN, MPNN, FP-GNN, HiGNN and FG-BERT to perform phenotype-based activity prediction, virtual screening and similarity search tasks. A significant limitation of many existing 2D graph-based models is their inability to directly incorporate 3D molecular geometry, which is crucial for predicting quantum chemical properties accurately. To address the problem of lacking 3D structural information, a GAT-based PointGAT model has been developed to predict quantum chemical properties considering 3D molecular geometry [45]. This model uses two basic feature extraction modules. The first module is for graph-level feature extraction with a modified Attentive FP, while the second module models geometric features through a point cloud methodology. Each atom is represented in the point cloud module by its relative 3D coordinates and additional features, such as charge, radius and ring information. The vectors obtained from these two modules are combined to produce final predictions. Relying on single molecular representations may not capture the full spectrum of information needed for a comprehensive and accurate molecular understanding. To create a more comprehensive and accurate molecular representation by leveraging multiple perspectives, a new method, multi-view molecular representation learning (MvMRL), combines different molecular representations, such as molecular fingerprints, molecular graphs and SMILES arrays [46]. MvMRL consists of a multiscale CNN-SE block that extracts local and global features from SMILES sequences. This GNN encoder learns multiscale features from molecular graphs, a Multilayer MLP that captures non-linear relationships from molecular fingerprints, and a pair of cross-attention mechanisms that integrate information from different views. Challenges persist with common molecular representations, such as the non-uniqueness issue associated with SMILES sequences and the difficulty of standard molecular graphs in capturing global molecular information. Addressing the problems of the non-uniqueness of SMILES sequences and the inability of molecular graphs to capture global information, a model called Molecular Sharing and Molecule-Specific Representations (MSSP) combines multiple fingerprint features with graph features encoded by GNN [47]. Modal alignment and fusion are achieved by mapping molecules to both molecular sharing and molecule-specific representation domains. Acquiring large labelled datasets for training deep learning models can be challenging and costly. Furthermore, traditional contrastive learning often requires data augmentation strategies that might not preserve essential molecular properties or introduce unwanted biases. Addressing the scarcity of labelled data and the limitations of augmentation-based contrastive methods, a new self-supervised learning method, Dual Graph Neural Networks Contrast Learning (DGCL), uses two GNNs, GIN and GAT, for molecular feature prediction [48]. This method applies contrast learning to extract different features of the same molecule. It preserves the molecule’s intrinsic properties, eliminating the data augmentation required in traditional methods. DGCL also combines representations from graphical network models pre-trained on downstream tasks with hybrid molecular fingerprints that provide functional group information. Furthermore, a limitation in some graph-based clustering approaches is separating the representation learning stage from the clustering process, which might lead to suboptimal cluster assignments. In contrast to existing approaches that separate representation learning and clustering, models such as FCGCN, developed using fuzzy logic integrated with the GCN model, address this [49]. FCGCN encodes the graph structure/node attributes with GCN and uses an MLP to directly estimate the fuzzy cluster membership of nodes in an end-to-end manner.

Predicting complex properties like pesticide similarity can benefit significantly from integrating multiple data modalities. Yet, many existing models are uni-modal or lack sufficient interpretability to understand the basis of their predictions. To address this need for multi-modal integration and improved interpretability, Pesti-DGI-Net, a new multi-modal deep learning architecture for pesticide similarity prediction, was developed [50]. The model simultaneously learns information from molecular graphs and fingerprints. A spatial GNN and an attention mechanism are used for molecular graphs, while molecule features are used to initialise nodes. Three complementary fingerprints, MACCS, Pharmacophore ErG and PubChem, are fed into a neural network model for fingerprint information. FP-GNN is a method that produces a final prediction by combining GNN and ANN outputs. Many existing methods struggle to effectively integrate 3D spatial structures and multi-level substructure information simultaneously for molecular feature prediction. To address this gap by integrating 3D spatial context with substructure details, 3DSGIMD, a novel method using a 3D spatial graph-focusing network and structure-based feature fusion, was developed [32]. This method integrates 3D spatial structures and substructure information of molecules at multiple levels. The model consists of two modules: a 3D Spatial Graph Focusing Network (3DSGFN) that uses EGNN to learn from 3D structures and a Molecular Descriptor Network (MDN) that extracts information from molecular descriptors derived from 2D molecules. The Graph Spatial Convolution Focusing Mechanism (GSCFM) within 3DSGFN combines 3D information, neighbourhood relationships and atomic environments to learn structural properties and determine the contribution of each atom to the predicted properties.

While the studies mentioned above have advanced molecular feature prediction by addressing specific limitations, such as incorporating hierarchical information, fusing multi-modal data, utilising 3D structures, or improving self-supervised learning, a persistent challenge remains: many approaches still rely on a single type of GNN architecture. However, different GNN architectures like GCNs, GATs and GINs possess distinct strengths and weaknesses. GCNs excel at capturing local topology efficiently but may overlook global patterns. GATs can focus on critical features via attention but can be computationally intensive. GINs offer strong theoretical discriminative power but might be sensitive to graph structure details. This reliance on individual architectures limits the model’s robustness and ability to capture the full complexity of molecular information, thus defining a significant research gap. Therefore, this study’s core scientific question is: Can a hybrid model combining the complementary capabilities of GCN, GAT, and GIN overcome the inherent limitations of using these architectures in isolation and achieve more accurate and generalisable molecular feature predictions? A novel hybrid GNN framework for molecular property classification is proposed to investigate this question. The central hypothesis is that a more comprehensive and robust molecular representation can be created by concurrently leveraging GCNs for local structural context, GATs for identifying salient features through attention and GINs for capturing fine-grained topological distinctions. The distinct feature sets learned by each parallel GNN branch are subsequently integrated, aiming to harness their synergistic potential. Following this feature fusion, a Bidirectional Long Short-Term Memory (BiLSTM) layer is employed to process the combined attributes. The inclusion of BiLSTM allows the model to capture potential sequential dependencies or relationships among the elements within the fused feature vector before generating the final classification output.

## Proposed method

Representing molecules as graphs allows for capturing intricate structural information, enabling graph-based deep learning methods to achieve high performance in property prediction tasks. However, as highlighted in the introduction, relying on a single type of Graph Neural Network (GNN) architecture often provides only one perspective on the molecular structure, potentially limiting model robustness and generalisation. While hybrid approaches combining graphical representations with other data forms exist, these strategies can introduce significant computational overhead and often require complex integration steps. Furthermore, applying distinct model types to different data modalities within the same framework can hinder a holistic evaluation of the molecular data. To circumvent these challenges while leveraging diverse graph perspectives, this work introduces Graph-Aware AURA-LSTM, a novel deep learning architecture designed for molecular feature classification.

**Fig. 1:**
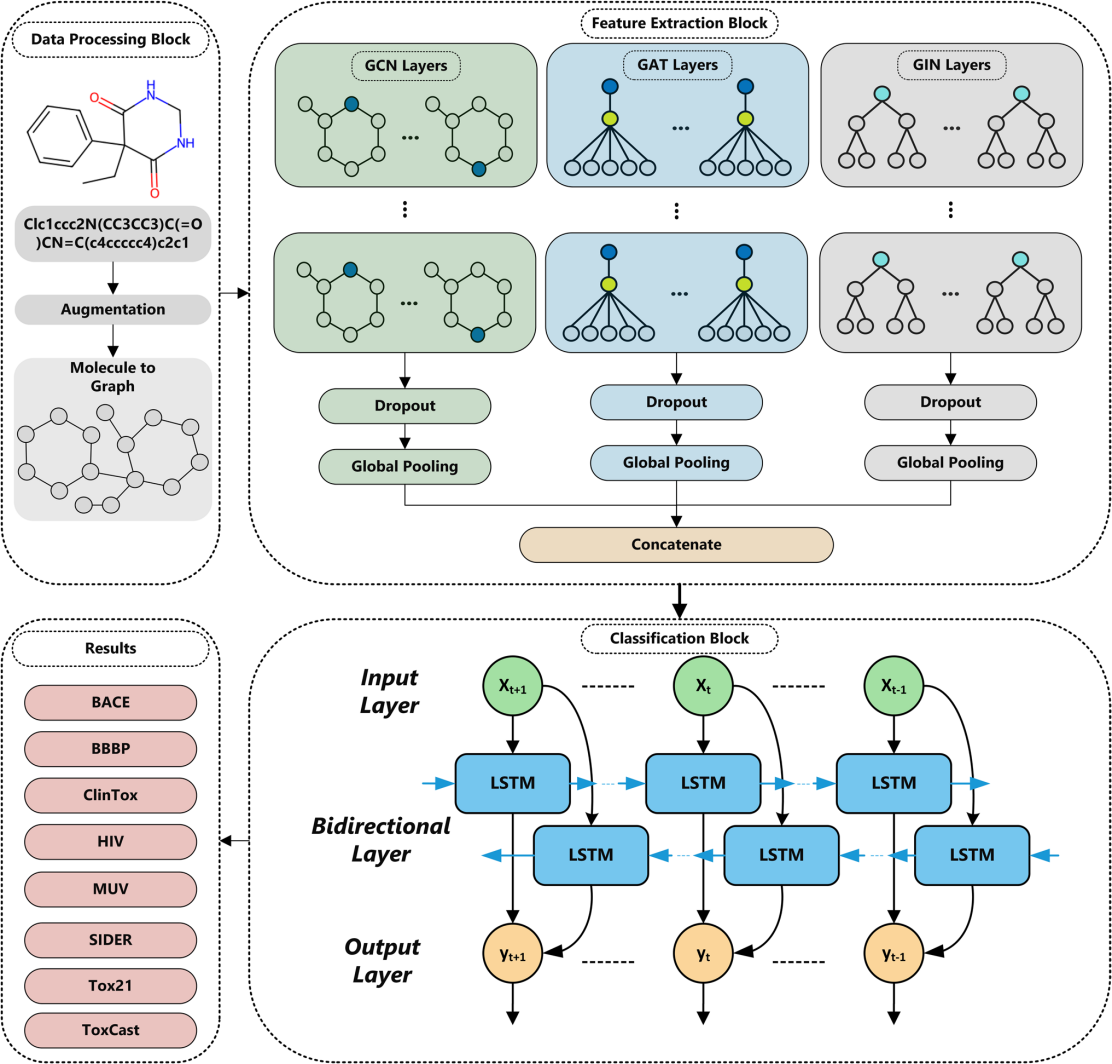
Workflow of Graph-Aware AURA-LSTM

Graph-Aware AURA-LSTM offers a novel approach to the challenges of processing and analysing complex graphical data, especially for high-performance classification of data containing irregular and heterogeneous structures. The method combines GAT, GCN and GIN architectures in parallel for feature extraction from the graphical representation of molecules. It leverages the strengths of each model, enabling the creation of richer and more robust feature matrices. The GAT architecture gives more weight to essential nodes, thanks to its attention mechanism, enabling more efficient use of the information obtained from neighbourhood relationships. At the same time, GCN combines local neighbourhood information to understand the overall structure of graphs and enables deeper representation learning. Conversely, GIN increases the model’s generalisation capability by using the isomorphic properties of graph structures and allows a better understanding of complex structural relationships in molecules. Molecular graphs are applied simultaneously used as input for these three models as input to obtain the feature matrix by considering different perspectives of the molecules. These GNN architectures extract features corresponding to various essential details from the molecular graphs given as input. At this stage, GNN architectures are used as a feature extraction block. The output matrix is obtained from the parallel GNN architectures with a single forward propagation. Therefore, there are no steps such as training and parameter optimisation in the GNN layers. This significantly reduces the computational burden of the proposed method. Then, the feature matrices obtained from these three architectures are fused and transmitted to the BiLSTM layer. BiLSTM allows for evaluating molecular information from different GNN architectures in sequential correlations, considering past and future contexts. It therefore provides a temporal learning mechanism from graph data. This integrated hybrid approach allows for efficient temporal and spatial information processing while improving the model’s overall performance, resulting in higher accuracy rates on complex datasets. The block diagram of the developed AURA-LSTM method is shown in Fig. 1.

### Dataset

Well-defined and widely used benchmark datasets have been used to evaluate and compare model performance. However, some molecular datasets contain low numbers of molecules, while others contain high numbers of molecules. In addition, the performance of the models cannot be measured at the desired level due to the unbalanced class distribution of the data. For this reason, it was preferred to use datasets containing various numbers of molecules to measure the performance of the AURA-LSTM method. In this study, benchmark datasets, which are especially important in the biomedical field, were used. In this context, MoleculeNet datasets were used to evaluate and compare the method’s overall performance [51]. Among the MoleculeNet datasets, the datasets in the Biophysics and Physiology sub-headings were preferred. The datasets used in the study are summarized in Table 1.

**Table 1:** Overview of the MoleculeNet benchmark datasets used in this study

Dataset	Description	Molecules
BACE	Prediction of binding results for a set of inhibitors of human β-secretase 1	1513
BBBP	Prediction of the blood–brain barrier permeability of molecules	2039
ClinTox	Prediction of clinical trial toxicity and FDA approval status	1477
HIV	Prediction of experimentally measured abilities to inhibit HIV replication	41,127
MUV	Prediction of a subset of PubChem BioAssay	93,087
SIDER	Prediction of marketed adverse drug reactions	1427
Tox21	Prediction of qualitative toxicity measurements on 12 biological targets	7831
ToxCast	Prediction of toxicology data for an extensive library of compounds based on in vitro screening	8575

As with biophysics datasets, datasets with classification problems were preferred. The BACE (β-secretase 1) dataset contains 1513 molecules to predict the inhibition activity of the enzyme β-secretase 1, a potential drug target for treating Alzheimer’s disease. It contains binary labels (active/inactive) indicating the molecules’ ability to inhibit BACE. The class distribution is 1039 inactive and 474 active molecules. The BACE dataset was used to evaluate the efficiency of predicting BACE inhibition activity. An HIV dataset containing 41,127 molecules was prepared to predict the replication of HIV molecules. Identifying molecules that inhibit HIV replication is of great importance in disease treatment. The dataset consists of 40,462 inactive and 665 active class labels. The MUV dataset is a dataset consisting of a total of 93,087 molecules, including multiple classification tasks. The “MUV-548” label, which contains the highest number of class labels, was classified among these classes.

On the other hand, Physiology datasets were also used in the study. The BBBP (Blood–Brain Barrier Permeability) dataset contains 2039 small molecules and aims to predict whether they can cross the blood–brain barrier (BBB). The BBB is a selective barrier that protects the brain from toxins and pathogens. The dataset contains binary labels (permeable/impermeable) indicating the BBB permeability of molecules, with a class distribution of 1202 permeable and 837 impermeable. The ClinTox dataset is designed to predict the clinically observed toxicity of drug candidates, containing 1478 molecules. The class distribution for FDA approval is 1318 approved and 160 unapproved; for clinical toxicity, 1356 non-toxic and 122 toxic. The clinically approved data tag was used for the classification task. The ToxCast dataset is a multiclass labelled dataset containing 8575 molecules. ToxCast contains toxicity data from high-throughput screening assays and 617 different toxicity pathways. As the class distribution differs in each toxicity pathway, it provides a wide range of estimates of the various toxicity effects of chemicals. Data with the label “ACEA_T47D_80hr_Negative” were used in this study. The SIDER dataset contains 1427 drugs and their 5868 side effect associations. Due to the multiclass structure of the side effects classification tasks, the frequency of side effects varies in the dataset. The Tox21 dataset contains 7831 molecules and 12 classification problems. In this study, data with the label “NR-ER” were used. This dataset was used to evaluate model performance in predicting the potential toxicity of environmental chemicals and drug candidates.

### SMILES-based augmentation

Molecules are unique. SMILES representations are a standard representation type that allows these molecules to be expressed as a one-dimensional array. However, SMILES representations of molecules can generate multiple valid SMILES strings for a single molecule, leading to duplicates and inconsistencies in datasets. Furthermore, SMILES strings may not accurately reflect the 3D structure and stereoisomerism of molecules, which prevents the expression of the variety of molecules available with this representation type [52]. These limitations negatively affect the performance of machine learning models, especially in low-data or data-imbalance classification scenarios. To overcome these problems, data augmentation techniques based on SMILES representations of molecules have been widely applied in the literature [53]. SMILES-based data augmentation adds additional examples to the dataset by generating structural variations that do not disrupt the chemical structure of a molecule, allowing the model to compensate for potential performance losses due to class imbalance and data insufficiency.

**Fig. 2:**
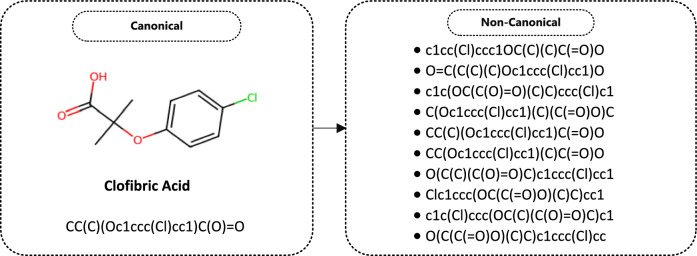
An example of a SMILES-based data augmentation result

This study applied the SMILES-based augmentation method to enable the model to achieve higher generalisation capacity to different molecular structures. Diversifying the SMILES variants of molecules found in all the datasets improves the prediction performance. It makes the model more robust by allowing it to cover a broader chemical space during model training. This study applied a fivefold data augmentation to the SMILES strings of molecules. Open-source libraries were used to implement this method. First, a SMILES string is applied as input to this method and converted into a molecule object. At this stage, non-canonical derivatives of the molecule were randomly generated. A control mechanism was added to prevent the generation of identical SMILES strings. At this stage, the data are divided into a certain number of folds using K-Fold and each data point is assigned a fold label. Thus, the original fold distribution is preserved after the data replication process. This method maintains data integrity by integrating variants into the original dataset, performing fold separation for k-fold cross-validation, and removing duplicates between the original and augmented data. This results in a more comprehensive, representative and diverse dataset for model training. In addition, the model makes more robust and reliable predictions by aiming to minimise the risk of overfitting. All datasets obtained from this process were divided into training and test data in a 70:30 ratio. An example of a SMILES-based data augmentation process implemented from the datasets used in the study is given in Fig. 2.

### Molecule to graph

Accurate prediction of the properties of molecules depends on the most efficient representation of chemical compounds. While molecular descriptors and fingerprints are generally preferred for one-dimensional representation, their representation in graphical form allows molecules to be effectively represented as they are found in nature. The graphical representation of molecules is a powerful way to express their chemical structure mathematically and has been widely used in deep learning models. Molecules consisting of atoms and chemical bonds can be easily represented in the framework of graph theory, where atoms are modelled as nodes as the set $V$ and chemical bonds as edges as the set $E$. In the first step, each atom is identified as a node by an attribute vector containing chemical information, such as chemical type, charge state, hybridisation state and binding properties. The prominent feature of this graphical representation is that it allows a detailed analysis of the chemical structure of molecules. The graphical representation of molecules makes it possible to effectively use GNN models, one of the deep learning methods, in molecular analyses. GNN models provide learning by considering the properties of each node and the neighbouring nodes and the relationships between them. This feature makes it possible to understand the interaction of an atom with other atoms in its environment and the effect of these interactions on bond types. The graphical representation of molecular structure allows the study of local and general relationships of molecules, enabling their chemical behaviour to be modelled. Thus, molecules’ structural properties, biological activities and intermolecular interactions can be analysed more systematically.

In this graphical representation method, each molecule’s structural organisation and chemical bonds are treated by mathematical modelling. The graph structure is created by placing atoms as nodes and representing bonds by edges. The feature vectors describe the chemical properties of the nodes $x_{i}$, while the edge properties $e_{i,j}$ contain information such as bond type and length so that the structural characteristics of the molecule can be analysed in detail. In this study, the graphical representation of molecules was performed in several stages. Firstly, the atoms of each molecule in the dataset are represented in the graph as nodes. Then, the chemical bonds between atoms $E\, = \,\{ e_{i,j} \left| {i,\,j\, \in V\} } \right.$ were added to the graph as an edge. Thus, the chemical species of atoms, bond states, charges, hybridisation state and atom type are carried through the nodes, while the edges allow the bond types to be examined. On the other hand, a symmetric adjacency matrix $A \in {\mathbb{R}}^{NxN}$ is used to represent the molecule. If there is a bond between $v_{i}$ and $v_{j}$ atoms of the molecule, $A_{i,j} = 1$, otherwise $A_{i,j} = 0$ if there is no bond. The proposed AURA-LSTM method aims to gain a deeper understanding of the graphical knowledge of molecules and to improve functional predictions of molecules. These graphical representations of molecules are applied as input to the designed parallel graphical networks. This approach expresses the general form of a molecule and the structure properties of the molecule are mathematically represented in the form $M = \left\{ {A,X,E} \right\}$. In this notation, $A$ is the adjacency matrix, $X$ is the attribute matrix of the features of the atoms and $E$ is the matrix of the edge features.

### Graph convolutional network

GNNs are a technique that has attracted great interest in recent years and offers a promising approach to analysing and interpreting data in non-Euclidean geometries compared to traditional machine learning methods. GNNs stand out as architectures with a high potential for information extraction, especially from graphically organised data. Compared to other deep learning methods, especially CNNs and Recurrent Neural Networks (RNNs), GNNs achieve highly effective results in addressing problems related to the invariant nature of graphs. GNNs can be defined as deep neural network architectures capable of learning node representations based on the local neighbourhood relationships of molecules, primarily those described as graphs. In this context, inputs to GNN networks usually consist of data such as graphical structures of molecules, coded neighbourhood matrices and edge features. GNNs can iteratively update the node representation by using the graph structure to obtain a feature representation of the nodes or graph; this update can be provided by convolutional processing or from node neighbourhoods. Thus, GNNs offer significant advantages in various application areas by efficiently processing complex relations and structured data.

The forward computation for GNN consists of repeating the message collection and update steps through the layers. In a graph expressed as $G = (V,E)$, for each node $v_{i} \in V$, the initial features are described and summed in a feature matrix with the $H^{0} \in {\mathbb{R}}^{Nxd}$ condition $H^{0} = \left[ {h_{1}^{0} ,h_{2}^{0} ,...,h_{N}^{0} } \right]$. Here, the vector contains the properties of the $ith$ node and is $d$ dimensional. Also, if the adjacency matrix of the graph is $A \in {\mathbb{R}}^{NxN}$, $A_{ij} = 1$ means that there is an edge between nodes $i$ and $j$. Fundamentally, in the traditional GNN architecture, the following equation expresses the aggregation process.

1 $$ m_{i}^{l + 1} = \sum\limits_{j \in N(i)} {f\left( {h_{i}^{l} ,h_{j}^{l} ,e_{ij} } \right)} $$

Here, it represents the neighbours of the node $v_{i}$. $f$ is the message function and $m_{i}^{l + 1}$ is the message collected for node $v_{i}$ as it passes through the $lth$ layer. Then, using the meshes collected from the neighbours, the features of each node are updated as follows:

2 $$ h_{i}^{l + 1} = \varphi (W^{l} .m_{i}^{l + 1} + b^{l} ), $$

where $h_{i}^{l + 1}$ is the updated feature of node $v_{i}$ in layer $l + 1$. $W^{l}$ is the weight matrix and transforms the node features. $b^{l}$ is the bias and $\varphi$ is the activation function. This process is repeated as the layers are added end-to-end and the weight functions are updated sequentially and this process is repeated sequentially. However, in the proposed AURA-LSTM method, backpropagation algorithms are not utilised for weight updates of GNN models. Therefore, the first calculated sum matrix is obtained as the model output matrix.

In GCN, convolution is applied to the input vectors. At this stage, the unit matrix $I$ is added to matrix $A$ for normalisation. Therefore, $\widetilde{A} = A + I$ matrix is obtained. GCN uses the normalised adjacency matrix to update the features. Therefore, the diagonal matrix, called the degree matrix $D$, is defined as follows:

3 $$ D_{ii} = \sum\limits_{j} {\widetilde{A}_{ij} } $$

In this way, the normalisation matrix is obtained as follows:

4 $$ \widehat{A} = D^{{ - \frac{1}{2}}} \widetilde{A}D^{{ - \frac{1}{2}}} $$

Here, $\widehat{A}$ allows node features to be updated according to the normalised adjacency matrix. In the proposed AURA-LSTM method, GCN is used as a feature extraction layer. Therefore, the output matrix of the GCN layer is expressed as $H^{l}$ and the transformation is performed with a single weight matrix $W$. When this process is used for feature extraction, it is not subjected to training. As a result, the output of the GCN block is calculated as follows:

5 $$ H^{l} = \widehat{A}H^{0} W $$

Here, $H^{l}$ is the output matrix produced by the GCN layer and represents the new feature matrix to be used by the model. The $W$ matrix is a weight matrix that is kept constant since no training is performed and its dimension is $dxd{\prime}$, where $d{\prime}$ represents the output dimension. In summary, the output matrix for an untrained GCN layer is obtained by multiplying the input features matrix by the normalised adjacency matrix and then transforming it by a weight matrix. Equation 5 provides a new representation of each node’s feature updated with information from neighbouring nodes and, when used as a layer for feature extraction, directly obtains the output matrix of the model.

### Graph isomorphism networks

GIN is a robust neural network architecture with an exceptional ability to capture graph structural information. This method, which uses essential mechanisms among neural network models for analysing data with graph structure, is designed to maximise the discriminability between graphs. First developed by Xu et al. in 2018, GIN is a method based on the Weisfeiler-Lehman (WL) graph isomorphism test [54]. This test is recognised as an essential criterion for evaluating the expressive power of GINs. In GIN, a multiset aggregation process is used at each layer, and then a learnable linear transformation is applied to the node features, allowing them to be updated recursively. In this way, it emphasises the maximum discriminative mechanism of graph representations, enabling high-performance results in classification and regression problems. The GIN architecture goes beyond the average or maximum pooling strategies of other GNN models, combining node features with an MLP. This approach allows it to transform different input graphs into unique embeddings using an injective aggregation function. This injective feature enables isomorphic graphs to be represented similarly, while non-isomorphic graphs can be represented distinctly.

The feature extraction strategy in GIN is similar to that of GNN. The feature update process in Eq. 2 is expressed as follows by adding a degree of importance.

6 $$ h_{i}^{l + 1} = (1 + \varepsilon ) \cdot h_{i}^{0} + \sum\limits_{j \in N(i)} {h_{j}^{0} } $$

Here, a scalar parameter (which can be fixed or learnable) increases or decreases the importance of the node’s attributes. This value is a weight given as a model parameter, allowing the node to update its attribute with discriminative information from neighbouring nodes. $h_{i}^{l}$ stands for the updated node feature vector. The resulting updated node features are passed through an MLP and obtained in the following form.

7 $$ h_{i}^{l} = MLP\left( {(1 + \varepsilon ) \cdot h_{i}^{0} + \sum\limits_{j \in N(i)} {h_{j}^{0} } } \right) $$

Here, the MLP layer updates the attributes of each node with a non-linear transformation. This process increases the discriminability of different graphical structures and contributes to the theoretical maximum discriminative power of the GIN.

### Graph attention networks

GATs are a special GNN architecture that develops node features based on gathering information from local neighbours. Unlike other GNN methods, it employs an attention mechanism that allows each node to take into account information from neighbouring nodes to varying degrees. GATs work by assigning attention weights to each node’s neighbours to update node features iteratively. These attention weights allow each node to assign different degrees of importance to different nodes in its neighbourhood using coefficients. These coefficients determine the weight of each neighbour’s influence on the node. Thus, each node has a more appropriate information-gathering process, and a more meaningful and detailed feature extraction is performed instead of considering all neighbours with equal weight as in other methods. Another advantage of GATs is the ability to generalise to new nodes during training without depending on the graph structure. This capability makes the model suitable for inductive learning tasks. This structure allows the model to learn appropriate attention coefficients for nodes not seen during training.

In the GAT architecture, for each node $i$, information from its neighbour nodes is weighted according to its importance. GAT calculates the attention coefficient for the link with neighbour node $j$ in several steps. Firstly, each node feature is subjected to a linear transformation with the range of the weight matrix $W = \in^{{dxd{\prime} }}$:

8 $$ h_{i}{\prime} = Wh_{i} $$

Here, $h_{i}{\prime}$ is the transformed feature vector of node $i$. Then, the matrix $e_{ij}$, which is the attention score between nodes $i$ and $j$, is calculated based on the transformed features of these two nodes as follows.

9 $$ e_{ij} = LeakyReLU(a^{T} \cdot \left[ {Wh_{i} \parallel Wh_{j} } \right]) $$

Here, $a \in {\mathbb{R}}^{{2d{\prime} }}$ is the learnable attention vector and $\parallel$ is the fusion operator. LeakyReLU is used to smooth the attention score at negative values. The attention scores are then normalised with respect to all neighbours and used to weigh the effects of neighbouring nodes. Therefore, the $e_{ij}$ scores are normalised with the softmax function to obtain the attention coefficients $\alpha_{ij}$.

10 $$ \alpha_{ij} = \frac{{\exp (e_{ij} )}}{{\sum\limits_{{k \in {\mathcal{N}}(i)}} {\exp } (e_{ik} )}} $$

Equation 10 allows neighbouring nodes to be weighted according to their importance. The GAT layers represent each node using a weighted sum of node features, which allows each node to pay more attention to features from its nearest and most important neighbours. This mechanism avoids information loss and enables more efficient aggregation of features by focussing only on the nodes that need attention when transferring information between all neighbouring nodes. Since these attention coefficients can be computed in parallel, the GAT architecture can operate efficiently on large graphs. The updated features of each node are then calculated by multiplying the attention scores by the features of its neighbours as follows.

11 $$ h_{i}^{1} = \varphi \left( {\sum\limits_{{j \in {\mathcal{N}}(i)}} {\alpha_{ij} } \cdot Wh_{j} } \right) $$

In the developed method, multi-head attention is used. In this case, when there are $K$ independent attention heads, the outputs from each head are combined to obtain the feature matrix as shown in the equation below.

12 $$ h_{i}^{1} = \left\| {_{k = 1}^{K} \varphi \left( {\sum\limits_{{j \in {\mathcal{N}}(i)}} {\alpha_{ij}^{(k)} } W^{(k)} h_{j} } \right)} \right. $$

As a result, the GAT architecture enables each node to learn the relevance of each node with respect to its neighbours and to update node features. The output of the model is a graph-level feature vector, which is used directly for feature extraction without training.

### Bidirectional long short-term memory

BiLSTM has been developed to overcome various shortcomings of RNN models, which are particularly prominent in sequential data processing. The recurrent structure of RNNs may be inadequate for modelling long-term dependencies, and vanishing gradients or exploding gradients may be encountered during training [55]. LSTM networks, on the other hand, can overcome these problems by effectively controlling the data flow through cell state and three basic gate blocks [56]. In this respect, LSTMs are compelling models for learning long-term dependencies in sequential data [57, 58]. The LSTM structure considers the previous and subsequent states of the data sequence by storing information in the cell state. The input, forget and output gates control the flow of information. The input gate decides which new information to add to current cell; the forget gate stores only meaningful information in the cell by removing redundant information and the output gate determines the extent to which the information in the cell is transferred to the next layer. Thanks to these features, LSTM has a wide range of applications in sequential data analysis. BiLSTM offers a more advanced learning strategy than the LSTM model by processing the data sequence in both forward and backward directions. This bidirectional processing capability allows the model to access past and future information at each time step, allowing for deeper learning of contextual relationships.

The BiLSTM structure used in this study provides a more robust representation of sequential features by combining the outputs from forward and backward LSTM layers. The context information provided by BiLSTM is particularly advantageous for learning complex dependencies in sequential data. Since the BiLSTM model handles sequential information in both directions, it can establish a strong connection between contextual features and relationships. Therefore, the combined feature matrix obtained from different GNN layers can be evaluated, allowing different connections to be established. By summing or integrating the forward and backward information obtained in the output of the model, a meaningful representation is created for each data sample.

**Fig. 3:**
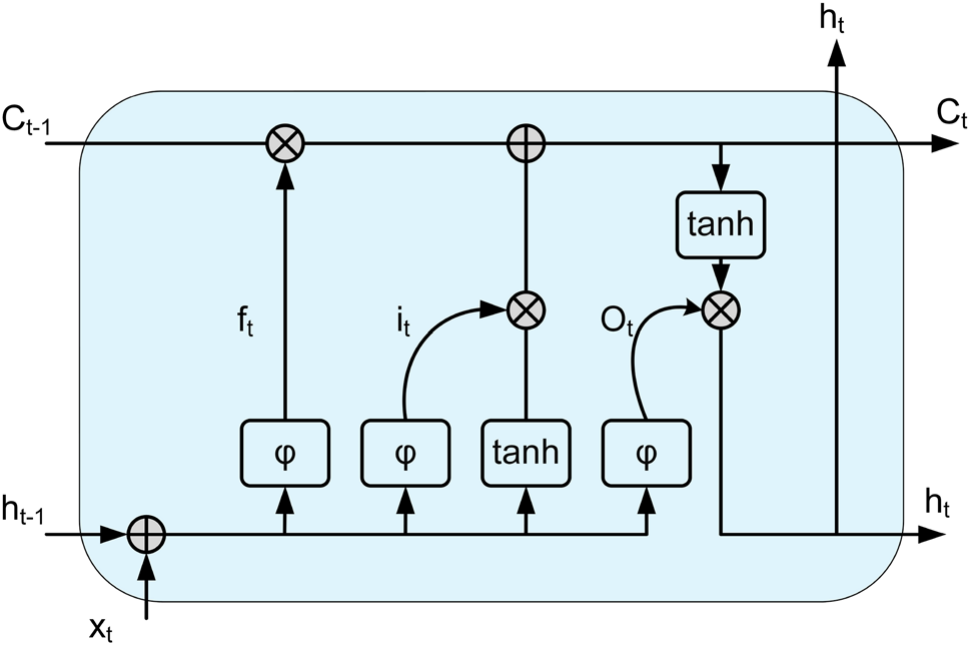
LSTM unit structure diagram

In this paper, text context characteristics are captured using BiLSTM. The forward LSTM and backward LSTM units comprise the BiLSTM unit, which gathers data in two opposing orientations. The LSTM unit is shown in Fig. 3.

The LSTM architecture has three gates; an input gate $i_{t}$, a forget gate $f_{t}$ and an output gate $o_{t}$. It also has a memory cell $C_{t}$ which enables it to learn long-distance dependencies in a sequence and a hidden state $h_{t}$. $\tilde{C}_{t}$ is candidate state value at time t of memory cell and is calculated by $\tanh$ function. The hidden state $h_{t}$ given input $x_{t}$ at time $t$ is computed as follows:

13 $$ \begin{gathered} i_{t} = \varphi (w_{i} \left[ {h_{t - 1} ,x_{t} } \right] + b_{i} ) \hfill \\ f_{t} = \varphi (w_{f} \left[ {h_{t - 1} ,x_{t} } \right] + b_{f} ) \hfill \\ \tilde{C}_{t} = \tanh (w_{c} \left[ {h_{t - 1} ,x_{t} } \right] + b_{c} ) \hfill \\ C_{t} = f_{t} \odot C_{t - 1} + i_{t} \odot \tilde{C}_{t} \hfill \\ o_{t} = \varphi (w_{o} \left[ {h_{t - 1} ,x_{t} } \right] + b_{o} ) \hfill \\ h_{t} = o_{t} \odot \tanh (C_{t} ) \hfill \\ \end{gathered}, $$

where $w_{f} ,w_{i} ,w_{c}$ and $w_{o}$ are the weight matrices, and $b_{f} ,b_{i} ,b_{c}$ and $b_{o}$ are the bias matrices. $\varphi$ denotes a logistic sigmoid function and $\odot$ denotes element-wise multiplication. $\tanh ( \bullet )$ is a hyperbolic tangent function. Since the features obtained from the GNN layers contain discrete values, the time variable is taken as 1. Two LSTMs are run simultaneously forward and backward on the input sequence in a bidirectional LSTM, a variation of the traditional LSTM. Future contextual information is captured using the forward LSTM, while previous contextual information is captured using the backward LSTM. The input sequences for our method come from the word embeddings of the opinionated sentences or the feature sequence extracted from the feature extraction layers. The final output of BiLSTM is the combined forward and backward output for each time step:

14 $$ h_{t}^{{{\mathrm{BiLSTM}}}} = \vec{h}_{t} ||\mathop{h}\limits^{\leftarrow} _{t} $$

In the equation, $\mathop{h}\limits^{\leftarrow} _{t}$ is the forward LSTM output and $\mathop{h}\limits^{\leftarrow} _{t}$ is the backward LSTM output.

## AURA-LSTM architecture

Using SMILES representations of the molecules, data augmentation was first applied. In this process step, the original class labels of the data were preserved. Then, the data obtained from different datasets were converted into graphical form. Then, the data in this graphical form were applied as input to the GIN, GCN and GAT models designed in parallel. The parallel design of these layers aims to capture the properties of molecules in different dimensions. In this way, a more generalised feature extraction step was provided. The feature matrices obtained from the three parallel GIN, GCN and GAT models were subjected to a feature fusion process. The purpose of this process is to combine the feature vectors learnt from the nodes of each model into a single feature matrix, thus providing a richer representation. After applying the pooling operator in the last layer of each model, the node feature vectors are obtained as follows for the GIN, GCN and GAT models:

15 $$ \begin{gathered} H_{{{\mathrm{GIN}}}} \in {\mathbb{R}}^{{N \times d_{{{\mathrm{GIN}}}} }} \hfill \\ H_{{{\mathrm{GCN}}}} \in {\mathbb{R}}^{{N \times d_{{{\mathrm{GCN}}}} }} \hfill \\ H_{{{\mathrm{GAT}}}} \in {\mathbb{R}}^{{N \times d_{{{\mathrm{GAT}}}} }} \hfill \\ \end{gathered} $$

Here, $N$ represents the number of data such as molecule graphs and $d_{{{\mathrm{GIN}}}} ,d_{{{\mathrm{GCN}}}} ,d_{{{\mathrm{GAT}}}}$ represents the feature dimension extracted in the last layer of each model. Combining these matrices forms a feature matrix merged along the feature dimension.

16 $$ H_{{{\mathrm{concat}}}} = {\mathrm{concat}}(H_{{{\mathrm{GIN}}}} ,H_{{{\mathrm{GCN}}}} ,H_{{{\mathrm{GAT}}}} ) $$

This operation combines matrices along feature dimensions, i.e.

17 $$ H_{{{\mathrm{concat}}}} \in {\mathbb{R}}^{{N \times (d_{{{\mathrm{GIN}}}} + d_{{{\mathrm{GCN}}}} + d_{{{\mathrm{GAT}}}} )}} $$

As a result, GIN can be expressed as a combination of feature vectors from GCN and GAT models:

18 $$ \begin{gathered} h_{concat}^{(i)} = \,\left[ {h_{GIN}^{(i)} \parallel h_{GCN}^{(i)} \parallel h_{GAT}^{(i)} } \right] \hfill \\ \hfill \\ \end{gathered} $$

Here, $h_{{{\mathrm{concat}}}}^{(i)} \in {\mathbb{R}}^{{d_{{{\mathrm{GIN}}}} + d_{{{\mathrm{GCN}}}} + d_{{{\mathrm{GAT}}}} }}$ stands for the fusion of vectors. Therefore, the matrix obtained by the feature fusion process in the AURA-LSTM method combines the features learnt by each model and enables a more comprehensive feature representation.

The combined feature matrix was then applied as input to the BiLSTM model. The BiLSTM model is preferred because it can learn the sequential relations on the feature matrix bidirectionally. This model performs feature extraction by considering the complex relationships and pipelines of molecules in both forward and backward directions, thus aiming to maximise classification performance. In addition, thanks to the high representation capacity of BiLSTM, long-term relationships in the data are captured more effectively.

The hyperparameters of the GIN, GCN and GAT layers used in the AURA-LSTM method are given in Table 2. GIN, GCN and GAT models operate only as a feature extraction block. Therefore, they do not contain any optimisation and backpropagation algorithms. Thus, the method reduces the computational costs considerably.

**Table 2:** Parameters of GNN-based architectures used in the AURA-LSTM model

Parameter	GAT Layer	GCN Layer	GIN Layer
Hidden Dimension-1	64	64	64
Hidden Dimension-2	32	32	32
Dropout Rate	0.3	0.2	0.3
Number of Heads	8	–	–
Activation	ReLU	ReLU	ReLU

The timesteps value of the BiLSTM model is set to 1 because the data are not time series, so each sample is processed independently. The 128 units in the first BiLSTM layer allow the model to learn more sequential relationships and features at a higher capacity. This number of units makes it possible to represent the input features in detail and to learn the relevant information with greater layer depth. The use of 64 units in the second BiLSTM layer optimises the model’s classification performance by extracting more abstract and high-level features. This structure allows the model to focus on more specific representations while reducing the information density. The dropout rate was set to 0.4 to avoid the problem of overfitting. Table 3 shows the hyperparameters of the BiLSTM model.

**Table 3:** BiLSTM model hyperparameters

Hyperparameters	Value
Units	128–64
Dropout	0.4
Output Layer	Sigmoid
Optimizer	Adam
Learning Rate	0.0005
Loss Function	Binary Crossentropy
Metric	AUC
Epoch	250
Batch Size	64

## Experimental results

In the AURA-LSTM method, three different GNN architectures, GIN, GAT and GCN, are connected in parallel and used as feature extraction layers. The BiLSTM layer was used to classify the features obtained from molecules in these layers. Therefore, thanks to this method designed to classify molecular features, it is possible to learn the features of molecules from many perspectives. In addition, whether there is a temporal connection between these graphical structures was measured through the BiLSTM unit. The performance of the proposed method was demonstrated in experiments on eight different MoleculeNet benchmark datasets. The datasets were randomly divided into training and validation in a 7:3 ratio. The proposed model was run under the same conditions for each dataset. All steps of the proposed method were implemented using Python-based open-source libraries. The experiments used a single NVIDIA GeForce GTX 3070 GPU machine with Intel Core i7-11700H CPU @ 4.90 GHz and 32 GB RAM. The GNN architectures were implemented only once in a forward propagation direction, so no backpropagation algorithms were used in the three GNN models. The training processes were carried out in the BiLSTM layer. ROC-AUC, PRC-AUC, recall and zero–one loss and metrics were used to measure the performance of the AURA-LSTM model. Although metrics obtained from training and validation data provide important information about the model’s generalisation ability in performance evaluation of deep learning models, it can be computationally challenging to measure the model’s performance on all data, especially in large datasets. To overcome this problem, performance metrics are calculated over the entire dataset using functions triggered at specific intervals or periods during model training. This allows for obtaining a more reliable estimate of models’ real-world performance. In addition, this method enables early detection of potential problems such as overfitting. In the scope of this study, these measurements were made at each epoch of each LSTM model to cover the entire dataset. In this way, the results’ reliability and the model’s success can be demonstrated more comprehensively. In addition, early stopping and dropout strategies were applied to prevent overfitting.

The unified approximation in the AURA-LSTM method allows molecules to be represented in a large space. This combined feature extraction layer allows the molecules to be evaluated differently. To demonstrate the effectiveness of this combined structure, the features extracted by each GNN model were classified separately. A series of experiments were conducted to measure and compare the contribution of each GNN architecture to the developed AURA-LSTM architecture. In these experiments, the features extracted from the GCN, GAT and GIN layers were classified separately in the BiLSTM layer. Then, the features obtained from these different GNN architectures were combined as GCN-GAT, GCN-GIN and GIN-GAT. The features obtained from these single and binary GNN models were classified in the BiLSTM layer, and their performances were measured. During the experiments, the GNN and BiLSTM model parameters were applied in the same way as for the AURA-LSTM model. In this way, a fair comparison could be made. Table 4 shows the training classification performances of the developed AURA-LSTM model for the GNN layers combined singly and doubly.

**Table 4:** Training performance results and comparative analysis of AURA-LSTM and its ablated variants

	Metric	BACE	BBBP	ClinTox	HIV	MUV	SIDER	Tox21	ToxCast
AURA-LSTM	AUC	0.9453	0.9647	0.9832	0.9354	0.8815	0.8296	0.9180	0.8566
	Loss	0.3121	0.2803	0.0943	0.1240	0.1784	0.3989	0.3514	0.4770
	Recall	0.8536	0.9061	0.9498	0.9215	0.8642	0.7405	0.8857	0.8197
	Zero–One Loss	0.1464	0.0939	0.0502	0.0144	0.1216	0.2595	0.1243	0.2153
GCN-BİLSTM	AUC	0.8054	0.8531	0.9574	0.8211	0.7356	0.6437	0.9132	0.8229
	Loss	0.5455	0.4778	0.2401	0.3022	0.2901	0.6691	0.3643	0.4601
	Recall	0.7488	0.8115	0.9285	0.7942	0.7235	0.6152	0.8650	0.7640
	Zero–One Loss	0.2512	0.1885	0.0715	0.2142	0.0812	0.3848	0.1256	0.2760
GAT-BİLSTM	AUC	0.9203	0.9397	0.9768	0.8467	0.7644	0.7636	0.8753	0.8518
	Loss	0.3729	0.3514	0.1857	0.3432	0.2568	0.5869	0.3764	0.5673
	Recall	0.8423	0.8774	0.9449	0.8111	0.7812	0.6934	0.7861	0.8240
	Zero–One Loss	0.1577	0.1226	0.0551	0.2355	0.1224	0.3066	0.1365	0.2460
GIN-BİLSTM	AUC	0.8567	0.8818	0.9576	0.8925	0.8411	0.6293	0.9142	0.8306
	Loss	0.4838	0.4466	0.2324	0.1954	0.1685	0.6694	0.3659	0.4512
	Recall	0.7753	0.8206	0.9285	0.8515	0.8012	0.5991	0.8551	0.7898
	Zero–One Loss	0.2247	0.1794	0.0715	0.0923	0.0944	0.4299	0.1253	0.3060
GCN-GAT-BİLSTM	AUC	0.9338	0.9102	0.9794	0.9014	0.8653	0.8035	0.8894	0.8488
	Loss	0.3465	0.4203	0.2014	0.1495	0.3576	0.5532	0.3771	0.5698
	Recall	0.8584	0.8458	0.9401	0.8841	0.8166	0.7214	0.8153	0.7440
	Zero–One Loss	0.1416	0.1542	0.0599	0.0964	0.0994	0.2786	0.1562	0.2810
GCN-GIN-BİLSTM	AUC	0.8674	0.9063	0.9644	0.8952	0.8145	0.6962	0.9186	0.8380
	Loss	0.4719	0.4024	0.2359	0.1785	0.1101	0.4455	0.3599	0.5615
	Recall	0.7951	0.8598	0.9285	0.8412	0.8107	0.6372	0.8051	0.8110
	Zero–One Loss	0.2049	0.1402	0.0715	0.1123	0.1235	0.3657	0.1025	0.2896
GIN-GAT-BİLSTM	AUC	0.9351	0.9369	0.9787	0.9153	0.8244	0.8095	0.8753	0.8495
	Loss	0.3368	0.3393	0.1624	0.1952	0.1667	0.5674	0.3772	0.5699
	Recall	0.8489	0.8605	0.9459	0.8852	0.7886	0.7464	0.8053	0.8210
	Zero–One Loss	0.1511	0.1395	0.0541	0.1001	0.1994	0.2625	0.1563	0.2321

When the training results obtained from the AURA-LSTM method are evaluated, it is seen that very successful results are obtained in terms of overall performance. It is shown through performance metrics that the model’s overall performance is remarkable in achieving high levels of accuracy and robustness. The AUC value of 0.9453 obtained in the BACE dataset shows that the model can successfully distinguish between positive and negative classes in this dataset. This success is supported by the model’s loss value of 0.3121 and Zero–One loss of 0.1464, indicating that the model’s predictions are generally reliable and have a low bias rate. The recall of 0.8536 in the BACE dataset suggests that the model can correctly identify many true positive samples. In the BBBP dataset, the AUC value reached a high level of 0.9647. The low loss value (0.2803) and Zero–One loss value (0.0939) of the model in this dataset indicate that its predictions are stable and consistent and have a low false classification rate. The 0.9061 recall value supports the model’s success in finding positive examples in the BBBP dataset and indicates that the model efficiently captures the unique features in the dataset. In the ClinTox dataset, the AUC value was 0.9832. The loss value (0.0943) and Zero–One loss value (0.0502) indicate that the model predicts the ClinTox dataset with high accuracy and misclassifies very few data points. The recall value was 0.9498, indicating that most positive cases were detected. The AUC value for the HIV dataset was 0.9354. The very low values of 0.1240 loss and 0.0144 Zero–One loss support the model’s performance on this dataset and indicate a high training accuracy. Furthermore, the recall value of 0.9215 shows the model’s ability to capture positive examples in this dataset. In the MUV dataset, an AUC value of 0.8815 was obtained. The 0.1784 loss value and 0.1216 Zero–One loss value indicate that the model’s predictions are generally accurate and work with a relatively low level of bias. The model showed a metric performance of 0.8296 AUC in the SIDER dataset. For the SIDER dataset, the loss value is 0.3989, and the Zero–One loss is 0.2595. Although these metrics indicate a slightly more challenging learning process compared to the other sets, the discrimination ability of the model is still present. In the Tox21 dataset, AURA-LSTM achieved a strong AUC value of 0.9180. The loss value was 0.3514, and the Zero–One loss value was 0.1243. These values show that the model learns effectively on this multi-task dataset. An AUC value of 0.8566 was obtained in the ToxCast dataset. Although the loss value of 0.4770 and Zero–One loss value of 0.2153 in this dataset reflect the difficulties encountered by the model, the AUC value shows that the classification performance is still satisfactory.

**Fig. 4:**
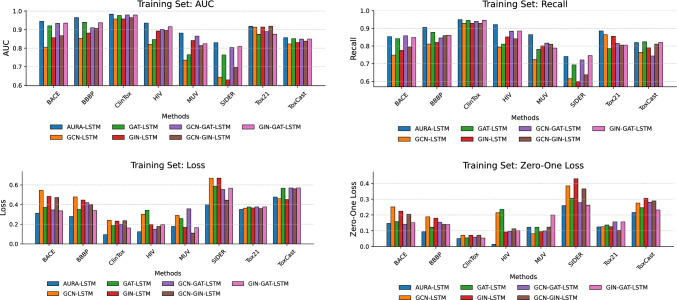
Comparative training performance of AURA-LSTM and its ablated versions

Furthermore, Table 4 presents the results of ablation studies to evaluate the performance of the proposed AURA-LSTM model and to understand the contribution of the different GNN layers on which it is based. The main objective of this comparison is to quantify the impact of the individual and combined use of GCN, GAT and GIN layers on a BiLSTM-based architecture and to demonstrate the advantages of AURA-LSTM. When the performances of the individual GNN architectures (GCN-BiLSTM, GAT-BiLSTM GIN-BiLSTM) are analysed, it can be seen that each of them performs with varying success on different datasets. While GAT-BiLSTM offers a relatively strong initial performance on some datasets, such as BACE (0.9203 AUC) and ToxCast (0.8518 AUC), GCN-BiLSTM generally shows lower performance results. GIN-BiLSTM achieved competitive results, especially on the HIV (0.8925 AUC) and MUV (0.8411 AUC) datasets, but significantly underperformed on some sets, such as SIDER (0.6293 AUC). These findings reveal that a single type of GNN may not be optimal for all tasks and each has its strengths and weaknesses in capturing different molecular structural features. Binary combinations of GNN layers generally show a tendency for improved performance compared to single GNN models. In particular, the GCN-GAT-BiLSTM and GIN-GAT-BiLSTM combinations significantly improved performance on some datasets over their constituent single models. This suggests that combining different GNN mechanisms can improve model performance by creating more unique and distinctive feature representations. However, the performance of the binary combinations also varies depending on the dataset, and a consistent superiority in all metrics has not yet been achieved. The proposed AURA-LSTM model consistently exhibits the highest AUC performance across all datasets evaluated. The AURA-LSTM model achieves the best AUC values in all datasets. This superiority is quite evident compared to single or binary GNN combinations. The high AUC values of AURA-LSTM are generally supported by lower loss and Zero–One loss values. At the same time, the high recall indicates the model’s effectiveness in detecting relevant positive samples with high accuracy. The comparative performance of all models and the superiority of AURA-LSTM in these metrics are visually summarised in Fig. 4. This ablation analysis strongly suggests combining different GNN layers, such as GCN, GAT and GIN, to create a synergistic effect. By leveraging the complementary feature extraction capabilities of each GNNs type, the AURA-LSTM architecture learns more comprehensive and robust molecular representations and efficiently processes these representations in terms of temporal dependencies with the BiLSTM layer. As a result, AURA-LSTM offers an overall and consistent performance advantage over models based on single or binary GNN combinations on the investigated molecular feature prediction tasks. These findings highlight the potential of varying and combining GNN architectures in complex molecular datasets.

The results of the validation tasks carried out to verify the performance trends observed during the training phase and to evaluate the generalisation capabilities of the models are presented in Table 5. In general, the validation metrics are consistent with the findings obtained during the training process. It is observed that the models, especially AURA-LSTM, primarily preserve the relative performance rankings observed in the training data. Although there are expected slight decreases in the validation metrics compared to the training results, this indicates that the models can generalise to new data without overfitting.

**Table 5:** Validation performance results and comparative analysis of AURA-LSTM and its ablated variants

	Metric	BACE	BBBP	ClinTox	HIV	MUV	SIDER	Tox21	ToxCast
AURA-LSTM	AUC	0.9194	0.9024	0.9736	0.9143	0.8655	0.8072	0.9156	0.8317
	Loss	0.3122	0.3155	0.2672	0.2345	0.2483	0.4604	0.3641	0.4420
	Recall	0.8423	0.8464	0.9407	0.9011	0.8501	0.7037	0.8999	0.7638
	Zero–One Loss	0.1577	0.1536	0.0966	0.0486	0.0216	0.3172	0.1025	0.2462
GCN-BİLSTM	AUC	0.7738	0.8708	0.9653	0.8354	0.7233	0.6295	0.9124	0.8207
	Loss	0.5937	0.4462	0.1861	0.2512	0.3237	0.5765	0.3723	0.4142
	Recall	0.6978	0.8399	0.9551	0.7825	0.7487	0.5967	0.8799	0.8107
	Zero–One Loss	0.3018	0.1601	0.0449	0.2358	0.0312	0.4033	0.1252	0.2338
GAT-BİLSTM	AUC	0.7937	0.9096	0.9574	0.8167	0.7543	0.6894	0.8702	0.8192
	Loss	0.6741	0.4008	0.2428	0.3962	0.2467	0.4890	0.3857	0.5687
	Recall	0.7004	0.8627	0.9281	0.7763	0.8711	0.7017	0.8204	0.7523
	Zero–One Loss	0.2996	0.1373	0.0719	0.2889	0.1350	0.3219	0.1352	0.2758
GIN-BİLSTM	AUC	0.8074	0.8626	0.9660	0.9052	0.8211	0.6804	0.7127	0.8269
	Loss	0.5453	0.4722	0.1903	0.1954	0.1985	0.4933	0.5835	0.5620
	Recall	0.7445	0.8039	0.9506	0.8554	0.7913	0.6711	0.6664	0.7862
	Zero–One Loss	0.2555	0.1961	0.0494	0.1134	0.1032	0.3289	0.3336	0.2560
GCN-GAT-BİLSTM	AUC	0.8201	0.9150	0.9491	0.9011	0.8345	0.7175	0.8614	0.8235
	Loss	0.6758	0.4290	0.2915	0.1348	0.1765	0.5954	0.3157	0.5706
	Recall	0.7829	0.8693	0.9169	0.8612	0.8211	0.7014	0.8104	0.7652
	Zero–One Loss	0.2247	0.1307	0.0831	0.1648	0.1088	0.2986	0.1896	0.2842
GCN-GIN-BİLSTM	AUC	0.8133	0.8815	0.9670	0.9005	0.8519	0.6367	0.9154	0.8287
	Loss	0.5588	0.4439	0.2050	0.1454	0.2222	0.4900	0.3717	0.5652
	Recall	0.7555	0.8213	0.9506	0.8683	0.8101	0.6131	0.8599	0.7874
	Zero–One Loss	0.2445	0.1683	0.0494	0.1256	0.1412	0.3869	0.1385	0.3120
GIN-GAT-BİLSTM	AUC	0.8203	0.9094	0.9538	0.8935	0.8111	0.7758	0.8702	0.8194
	Loss	0.6964	0.3917	0.2647	0.2133	0.1978	0.5904	0.3872	0.4652
	Recall	0.7357	0.8676	0.9303	0.8423	0.7752	0.5904	0.8304	0.7585
	Zero–One Loss	0.2643	0.1324	0.0697	0.1253	0.2114	0.4219	0.1085	0.2698

**Fig. 5:**
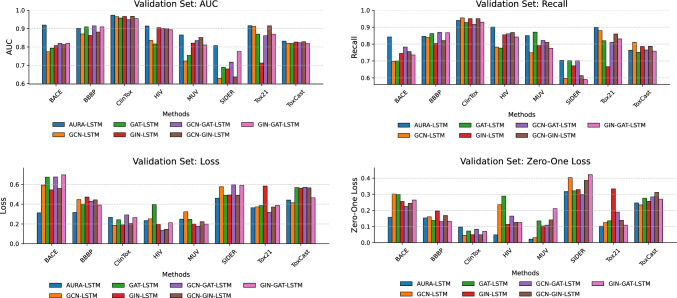
Comparative validation performance of AURA-LSTM and its ablated versions

Among the models evaluated in the ablation study, AURA-LSTM continued to perform the strongest in the validation sets. Compared to single (GCN, GAT, GIN) or binary GNN combinations, AURA-LSTM achieved the highest AUC values in all BACE (0.9194), BBBP (0.9024), ClinTox (0.9736), HIV (0.9143), MUV (0.8655), SIDER (0.8072), Tox21 (0.9156) and ToxCast (0.8317) datasets. The other metrics, loss, recall and Zero–One loss also generally support the superiority and balanced performance of AURA-LSTM. These validation results confirm the robustness of the AURA-LSTM architecture, which combines GCN, GAT and GIN layers, and its capacity for effective generalization over different molecular datasets. The model also demonstrated its ability to make successful predictions on previously unseen data. Figure 5 shows the comparative performance of all models during the validation phase.

**Fig. 6:**
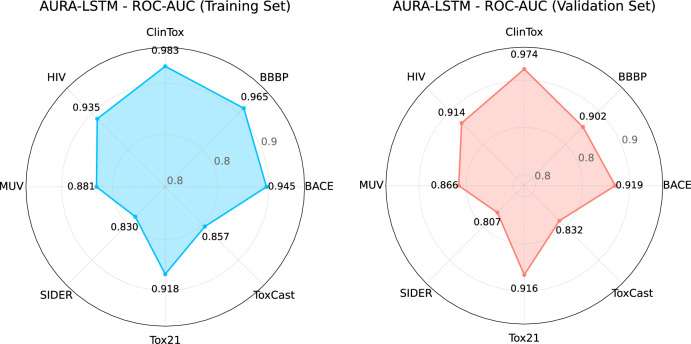
Radar charts of training and validation AUC values for the AURA-LSTM

Figure 6 provides radar plots summarising the AUC performance of the proposed AURA-LSTM model on different datasets for both the training and validation phases. In these plots, each axis represents one of the eight different datasets, and the distance of the point on the axis from the centre indicates the AUC score obtained by AURA-LSTM on that data set. The radar plot of the training step visually demonstrates the high AUC values obtained by AURA-LSTM on all datasets. It shows that the model has a very successful performance profile on the training data in general by covering a large area in the graph. When the validation phase graph is analysed, there is an expected slight decrease in the AUC values compared to the training results. This reflects the naturally occurring decline in the model’s performance when generalising. However, it is clear that AURA-LSTM maintains high AUC values in all datasets in the validation set and continues to exhibit a strong performance profile. These radar plots succinctly visualise the performance distribution of the AURA-LSTM model across different tasks and its generalizability from training to validation.

In addition to the training and evaluation performances, computational efficiency is critical in evaluating the AURA-LSTM method for practical applications. A notable aspect of the AURA-LSTM method is the low computational time observed, especially in the prediction phase. The main reason for this in the AURA-LSTM method is that GNN models are only treated as feature extraction blocks. The time complexity analysis allows a detailed analysis of the computational performance of the AURA-LSTM model based on the data in the presented timetable. The evaluation includes LSTM training time, LSTM test set prediction time, average prediction time per molecule and inference time of GNN models, such as GAT, GCN and GIN, which are included for comparison purposes and aim to quantify the model’s time cost in practical applications. Table 6 shows the time values of the computational steps.

**Table 6:** AURA-LSTM time complexity evaluation

Metric	BACE	BBBP	ClinTox	HIV	MUV	SIDER	Tox21	ToxCast
GAT Layer (Train Set) (s)	0.64	0.76	1.07	6.30	1.11	1.94	1.12	0.72
GAT Layer (Test Set) (s)	0.09	0.14	0.12	1.61	0.34	0.66	0.31	0.11
GCN Layer (Train Set) (s)	0.21	0.29	0.25	3.02	0.53	1.22	0.53	0.25
GCN Layer (Test Set) (s)	0.07	0.10	0.08	0.99	0.20	0.41	0.20	0.11
GIN Layer (Train Set) (s)	0.37	0.46	0.38	4.60	0.98	1.41	0.77	0.36
GIN Layer (Test Set) (s)	0.09	0.13	0.10	1.98	0.38	0.43	0.77	0.11
LSTM Training (s) (250 Epochs)	49.23	62.64	29.51	195.67	75.44	29.86	23.71	25.80
LSTM Prediction (Test Set) (s)	1.03	0.90	0.77	2.85	4.35	0.79	1.01	0.78
Avg. Prediction Time (ms/mol)	2.2641	1.4630	1.7240	1.3606	1.3040	1.9010	1.5289	1.5083

Table 6 shows that the training time of the AURA-LSTM model for 250 epochs is the most time-consuming step in developing the model. This time varies significantly from 23.71 s (Tox21) to 195.67 s (HIV), depending on the dataset used. This reflects that parameter optimisation is a computationally intensive process, which is expected due to the nature of deep learning models. The main reason for the time differences between the datasets is the differences in the number of data and the length of the SMILES strings given in Table 1. It should be noted that this training time is a one-time initial cost that is not repeated during the operational use of the model. The model’s prediction performance is significantly faster than the training time. The total LSTM prediction times over the entire test set range from 0.77 s (ClinTox) to 4.35 s (MUV), depending on the dataset. When the average prediction time per molecule, a more meaningful metric for practical use cases, is analysed, the AURA-LSTM model runs between 1.30 ms/mol and 2.26 ms/mol for all datasets. These values indicate that the model has a high processing speed for individual predictions. Once trained, the model can make inferences on the whole test dataset within reasonable times and is relatively fast per molecule. The observed per-molecule prediction times on the order of milliseconds suggest that the model may be a computationally favourable choice, especially for high-volume tasks, such as screening large molecule libraries or applications requiring fast response times.

## Discussion and conclusion

The effectiveness of the developed AURA-LSTM model in molecular feature classification is more clearly understood when it is evaluated not only by its intrinsic performance metrics but also in comparison with established methods in the existing literature. This comparative evaluation is critical to demonstrate the model’s practical value and potential advantages. In this context, to evaluate the performance of AURA-LSTM side by side with leading deep learning models, the results obtained on standard benchmark datasets using the widely accepted ROC-AUC and PRC-AUC metrics are compiled in Table 7.

**Table 7:** Performance comparison of AURA-LSTM’s ROC-AUC and PCR-AUC metrics with state-ofthe-art models

	BACE	BBBP	ClinTox	HIV	MUV	SIDER	Tox21	ToxCast
Attentive FP [59]	0.850	0.920	0.940	0.832	0.843	0.637	0.858	0.805
HRGCN + [60]	0.891	0.926	0.899	0.824	–	0.641	0.848	0.793
FP-GNN [61]	0.860	0.916	0.840	0.824	0.090	0.661	0.815	-
D-MPNN [62]	0.878	0.913	0.894	0.816	0.122	0.646	0.845	0.737
MoleculeNet (Graph) [51]	0.806	0.690	0.832	0.763	0.109	0.638	0.829	0.742
DLF-MFF [20]	0.868	0.884	0.831	–	–	–	–	–
TransFoxMol [63]	0.882	0.919	0.942	–	-	0.651	0.823	–
Uni-Mol [64]	0.857	0.729	0.919	0.808	0.821	0.659	0.796	0.696
DGCL [48]	0.914	0.737	0.971	0.814	0.781		0.771	–
MolCLR [65]	0.824	0.722	0.912	0.781	0.840	0.639	0.759	–
MvMRL [46]	0.891	0.962	0.975	–	–	0.653	0.845	–
TrimNet [66]	0.878	0.850	0.948	0.804	0.851	0.657	0.860	0.777
DMPNN [67]	0.798	0.867	0.857	–	–	0.568	0.784	–
Grover [68]	0.853	0.885	0.860	–	–	0.606	0.797	0.737
PremuNet [40]	0.843	0.733	**0.992**	–	–	0.628	0.740	–
Mole-BERT [69]	0.797	0.690	0.789	0.782	0.786	0.628	0.768	0.643
MCGNN [70]	0.892	0.933	0.945			0.639	0.836	0.744
GALLON [71]	0.804	0.723	0.991	0.753	–	–	–	–
GEM [72]	0.856	0.724	0.901	0.806	0.817	0.672	0.781	0.692
MSSP [47]	0.931	0.932	0.941					
3DSGIMD [32]	0.920	0.854	0.966	0.819		0.822	0.841	0.853
HiPM [73]	–	-	0.928		-	0.672	0.843	0.786
AEGNN-M [41]	0.867	0.941	0.996	0.794	0.802	**0.837**	0.849	–
AURA-LSTM	**0.945**	**0.964**	0.983	**0.935**	**0.881**	0.829	**0.918**	**0.856**

The best-performing results are highlighted in bold

The Graph-Aware AURA-LSTM model shows a remarkable performance improvement compared to other methods developed for molecular feature prediction models. The most important reason underlying this performance is that the AURA-LSTM method is able to represent molecules spatially in a robust way and to evaluate these representations by considering temporal relationships. When the state-of-art models comparing table is analysed, it is seen that the success rates of AURA-LSTM are higher in almost all datasets, indicating that the method has the potential for effective modelling in a wide range of applications. The ROC-AUC of 0.945 in the BACE dataset shows that AURA-LSTM successfully predicts highly complex and challenging molecules regarding biological activity. Compared to advanced models such as MvMRL (0.891) [46] and MCGNN (0.892) [70], the performance of AURA-LSTM demonstrates the more effective modelling capabilities of its neural network structure in molecular biology. The proposed model provided a performance improvement of 5.9% compared to the 0.892 value of MCGNN. In this context, the model offers the ability to predict the biological activity of molecular structures with high accuracy in biotechnological and pharmaceutical research. The fact that AURA-LSTM has a value of 0.964 in the BBBP dataset reveals that it is more successful than many other models in the literature in predicting the blood–brain barrier crossing potential. This analysis of the BBBP dataset, which has critical importance in neuropharmacology, emphasises the effectiveness of the deep learning architecture of AURA-LSTM compared to models such as MvMRL (0.962) and MCGNN (0.933). In this context, it shows a very successful classification performance, especially in solving a problem involving small molecules such as the BBBP dataset. AURA-LSTM, which reaches a success rate of 0.983 in the ClinTox dataset, provides impressive results regarding toxicity classification. In the academic literature, toxicity prediction is recognised as a fundamental research area for preclinical studies. Although models such as AEGNN-M (0.996) and PremuNet (0.992) achieve similar high accuracy rates in this dataset, the overall accuracy and stability provided by AURA-LSTM make it a model that will expand the scope of toxicological research. The HIV dataset is of significant academic importance in predicting molecules for HIV inhibition, and the ROC-AUC accuracy of AURA-LSTM of 0.935 emphasises its success in this area. Compared to Attentive FP (0.832) [41] and HRGCN + (0.824) [60], which are other prominent models in HIV research, the accuracy provided by AURA-LSTM allows more detailed predictions of HIV inhibition from biological data. AURA-LSTM exceeds the 0.832 performance of Attentive FP by 12.4% with 0.935. The performance result for this dataset shows that AURA-LSTM makes an innovative contribution to molecular biology and biotechnology research. With a PRC-AUC success rate of 0.871 on the MUV dataset, the accuracy achieved by AURA-LSTM, especially when compared to underperforming models such as FP-GNN (0.090) [61] and D-MPNN (0.122) [62], shows that AURA-LSTM provides a robust learning model against data insufficiency. The SIDER dataset is essential for predicting side effects, and AURA-LSTM’s accuracy of 0.829 can produce successful results in predicting side effects. AURA-LSTM outperforms models such as TrimNet (0.657) [66] and MolCLR (0.639) [65] in this area, offering a comprehensive research potential in the pharmaceutical field. With a success rate of 0.918 on the Tox21 dataset, AURA-LSTM outperformed models such as Attentive FP (0.858) [59] and TrimNet (0.860) in toxicity prediction. In the Tox21 dataset, the 0.918 accuracy rate exceeds the 0.860 rate of TrimNet, one of its closest competitors, by 6.7%. Since toxicity prediction has a wide range of applications in chemistry and biology, the accuracy of AURA-LSTM provides essential data for academic studies in this field. It is considered a robust model that can be used in toxicological analyses. The success rate of 0.856 obtained in the ToxCast dataset shows the ability of AURA-LSTM to evaluate the environmental and biological effects of chemical compounds. The high accuracy of AURA-LSTM compared to models such as Mole-BERT (0.643) [69] and Grover (0.737) [68] contributes to academic research in the field of environmental sciences and ecotoxicology. Overall, AURA-LSTM represents a new level of molecular property prediction in the academic literature. Its outstanding performance on different biomedical and chemical datasets shows the model offers a valid solution for both data-intensive applications and those with limited data. In this sense, AURA-LSTM demonstrates a broad research generalisation capability and classification performance in molecular feature prediction studies.

The Graph-Aware AURA-LSTM model proposed in this study provides significant advantages by addressing the limitations encountered in classifying molecular properties from an efficient and generalised perspective. In-depth analysis of graphical representations of molecules and versatile feature extraction play a critical role in the development of this method. The parallel use of GNN derivatives, especially GAT, GCN and GIN models, combining the strengths of each model, has allowed molecules to be evaluated from multiple perspectives rather than just a single one. GCN, which is used to understand the overall structure of graphical data; GAT, which focuses on the information obtained from neighbourhood relationships; and GIN, which considers the isomorphic relationships of molecular structures, are the most fundamental critical methods to the versatile structure of this architecture. This hybrid model, based on graphical representations of molecules, combines the strengths of each architecture in parallel and thus overcomes the limitations of a single architecture. GCNs effectively capture local structural information in molecular graphs and contribute to our model’s modelling of spatial relationships while providing computational efficiency. GATs, thanks to their attention mechanism, perform well in identifying critical functional groups and long-range interactions by focussing on specific structural features of molecules and complementing GCNs. GINs, on the other hand, capture fine details in molecular graphs, thanks to their ability to distinguish graph isomorphism and play a critical role in the temporal structure of our model to analyse the temporal evolution of molecules. The parallel use of these three GNN architectures enables AURA-LSTM to generate richer feature matrices than other methods in the literature, thus achieving higher accuracy rates. Four different performance metrics were utilised in the experiments using eight different benchmark datasets. The results show that AURA-LSTM significantly improves the molecular property classification performance. A comparison with molecular feature classification studies in the literature indicates that the AURA-LSTM method produces successful and comprehensive results compared to many other methods. It is observed that AURA-LSTM exhibits performance increases ranging from 2 to 9% compared to the most influential models in the literature. In this respect, the proposed method is innovative and successful for molecular feature classification. As a result, the hybrid model we developed allows us to produce more accurate predictions by weighting or combining the contribution of each architecture. AURA-LSTM provides a temporal learning mechanism from graph-based data, enabling efficient processing of spatial and temporal relationships and improving the model’s overall performance, achieving higher accuracy rates on complex datasets. Integrating the advantages of these models that study different aspects of molecules, AURA-LSTM offers a pioneering method for next-generation deep learning-based solutions in bioinformatics, chemistry and pharmacology. This architecture enables comprehensive analyses and reliable predictions in molecular feature classification studies with high accuracy rates in various datasets while reducing the computational burden.

In conclusion, integrating graph-based deep learning techniques, particularly GNNs such as GCNs, GATs and GINs, has recently driven a significant paradigm shift in molecular property prediction, drug repurposing and drug–drug interaction prediction. These models have demonstrated considerable potential by effectively learning from complex molecular graph structures, leading to more accurate predictions of molecular properties, adverse drug reactions and synergistic combinations, with a growing trend towards hybrid frameworks that leverage multiple GNNs variants for richer molecular representations. However, despite these advancements, critical challenges persist. The reliance on single GNN architectures often results in incomplete representations by specialising in only certain structural aspects, a limitation particularly detrimental in drug–drug interaction and repurposing tasks where subtle structural variations can profoundly alter biological outcomes. Furthermore, the constrained interpretability of current GNNs models impedes clinical trust and practical implementation. At the same time, the effective integration of heterogeneous features from multiple graph encoders without losing semantic context remains a formidable obstacle. These gaps underscore the need for more comprehensive, context-aware and interpretable architectures tailored to drug-related prediction tasks. Therefore, future work should focus on developing sophisticated hybrid GNNs architectures capable of synergistically combining the complementary strengths of diverse models, establishing robust methods to enhance model interpretability and build clinical trust, and designing innovative strategies for the seamless integration of heterogeneous features, ultimately aiming to improve predictive performance and facilitate the translation of these powerful tools into practical applications.

## Data Availability

All datasets analysed in this manuscript are publicly available. The MoleculeNet datasets can be accessed at the following link: https://moleculenet.org/datasets-1. The source code is available from GitHub at https://github.com/pala2515/AURA-LSTM
